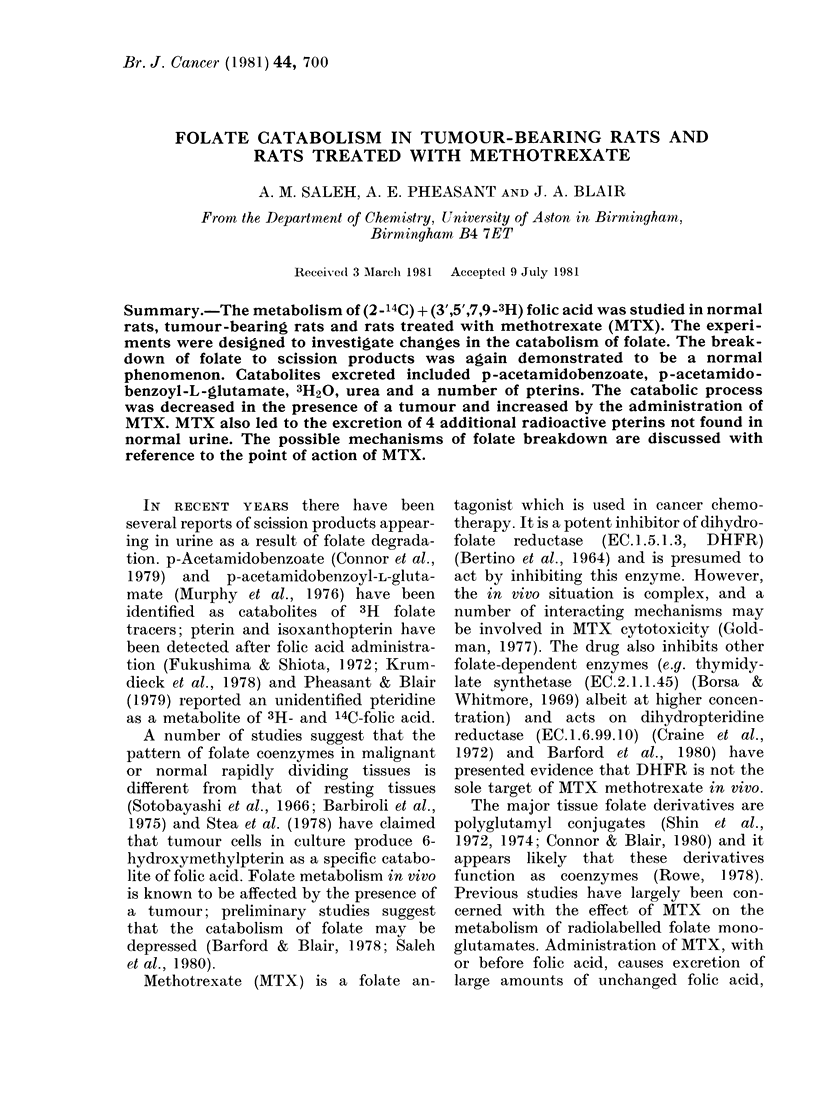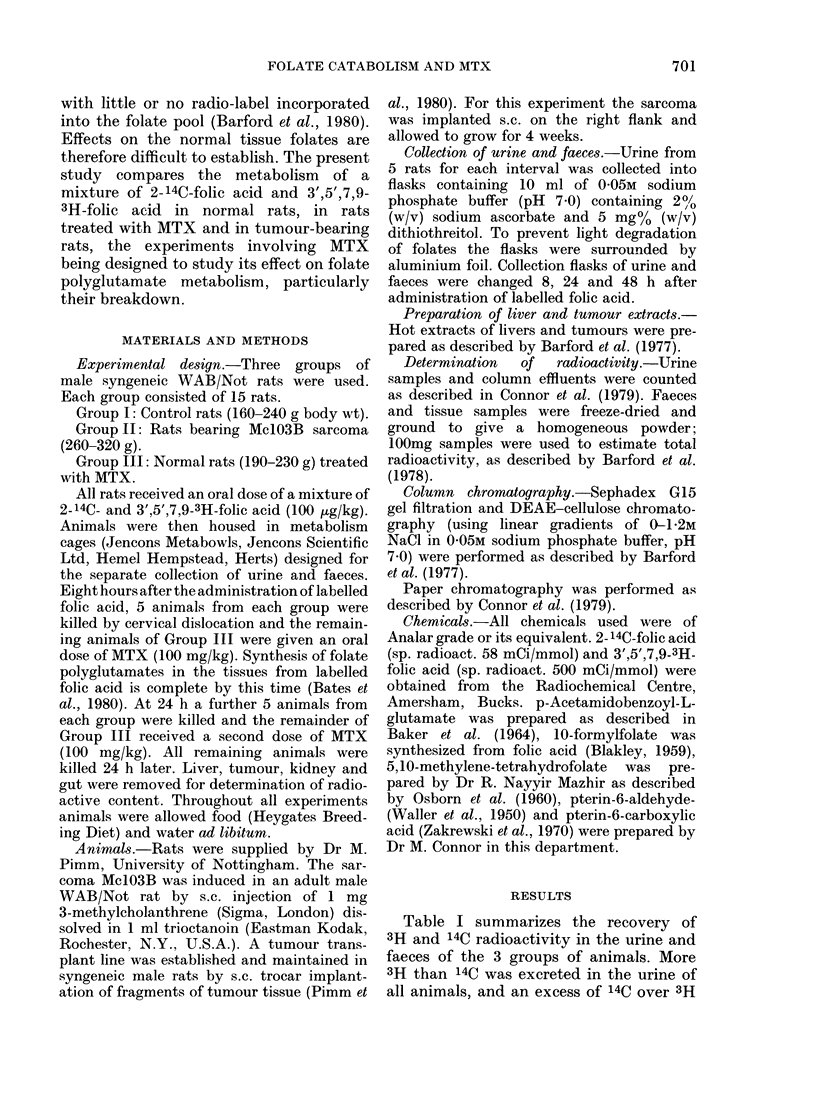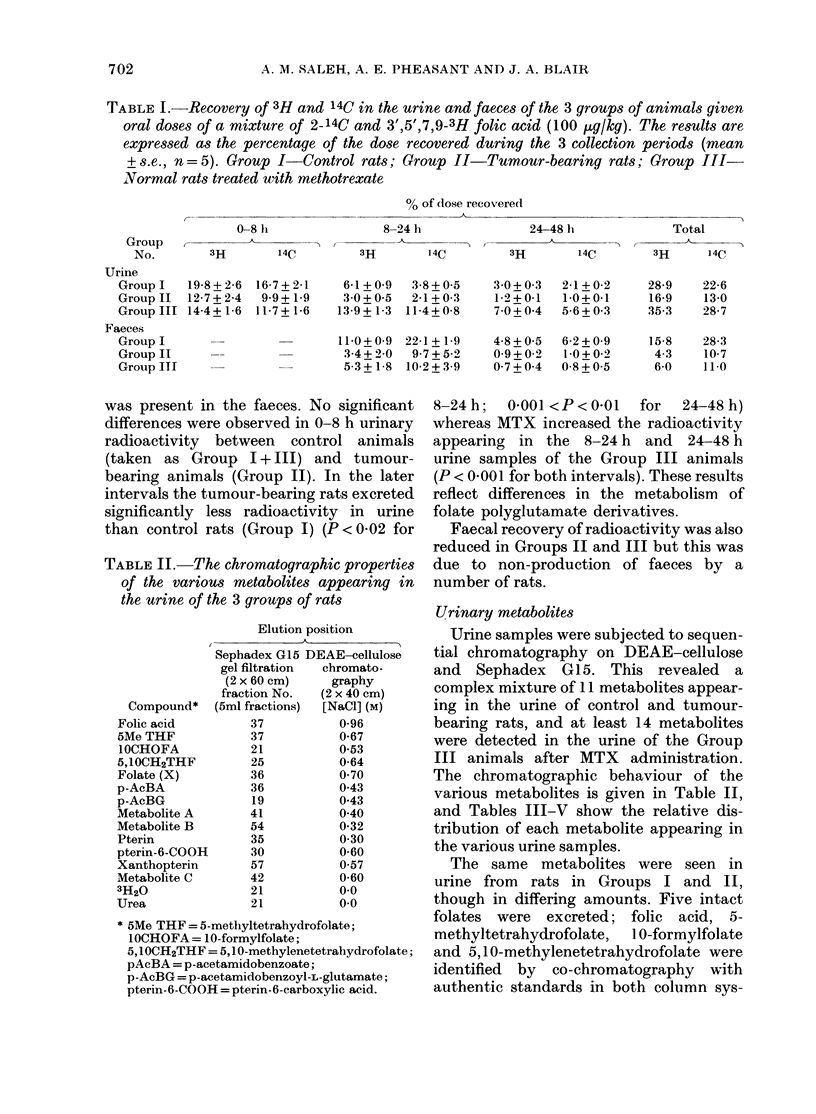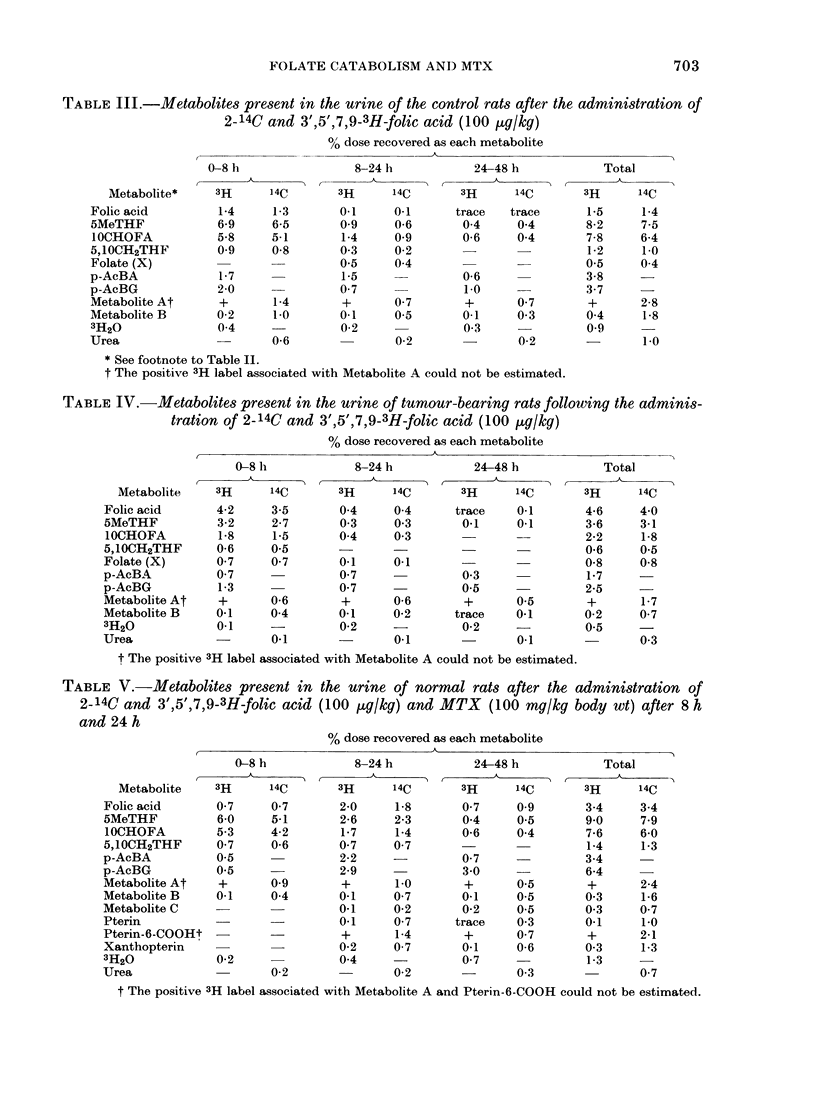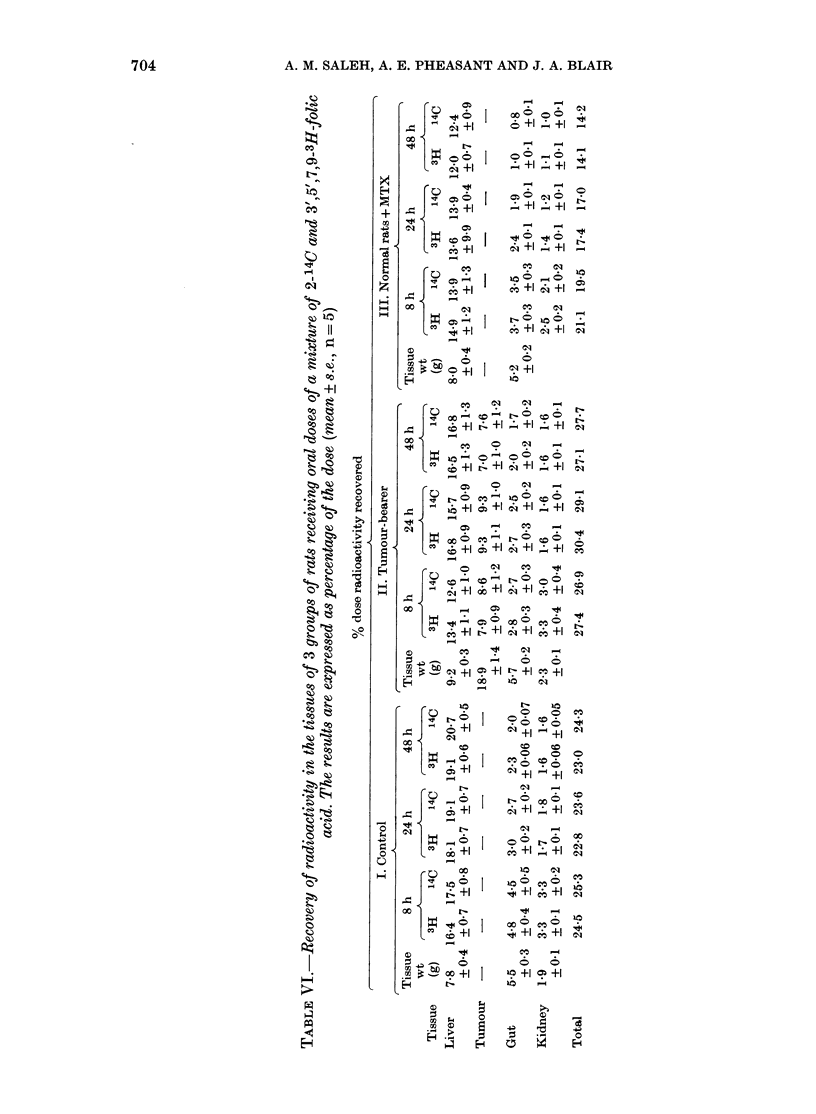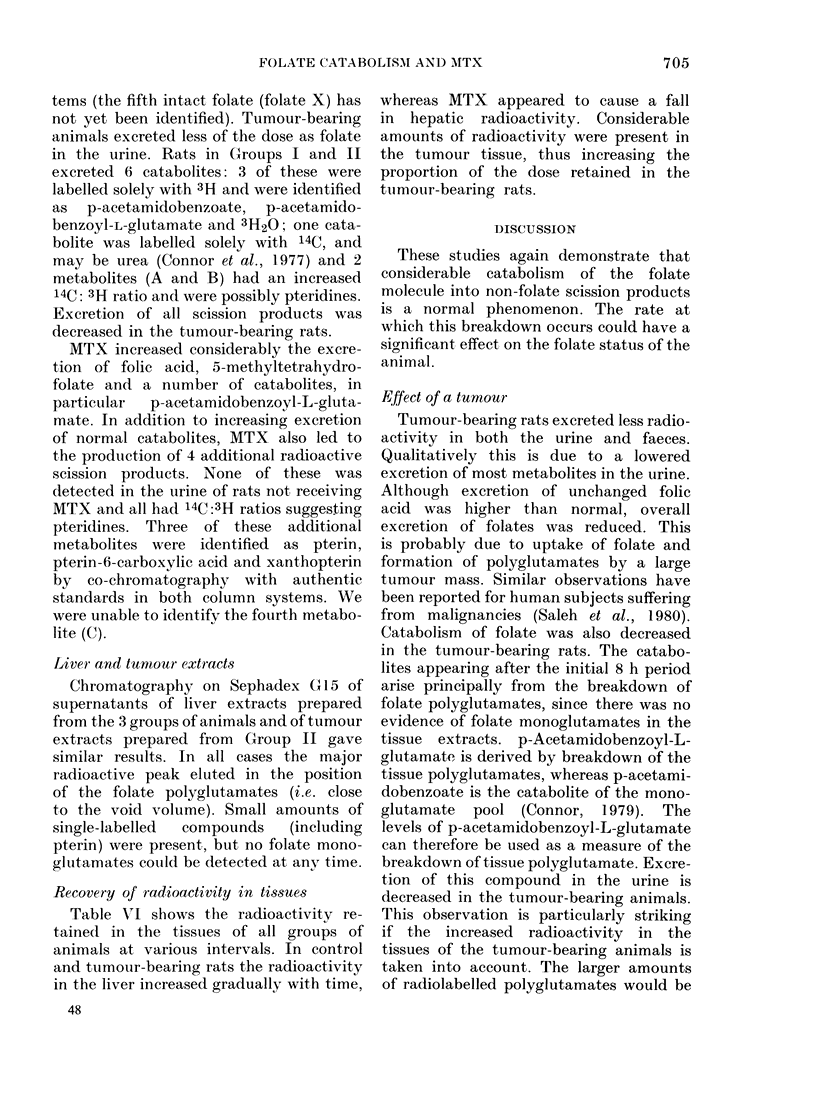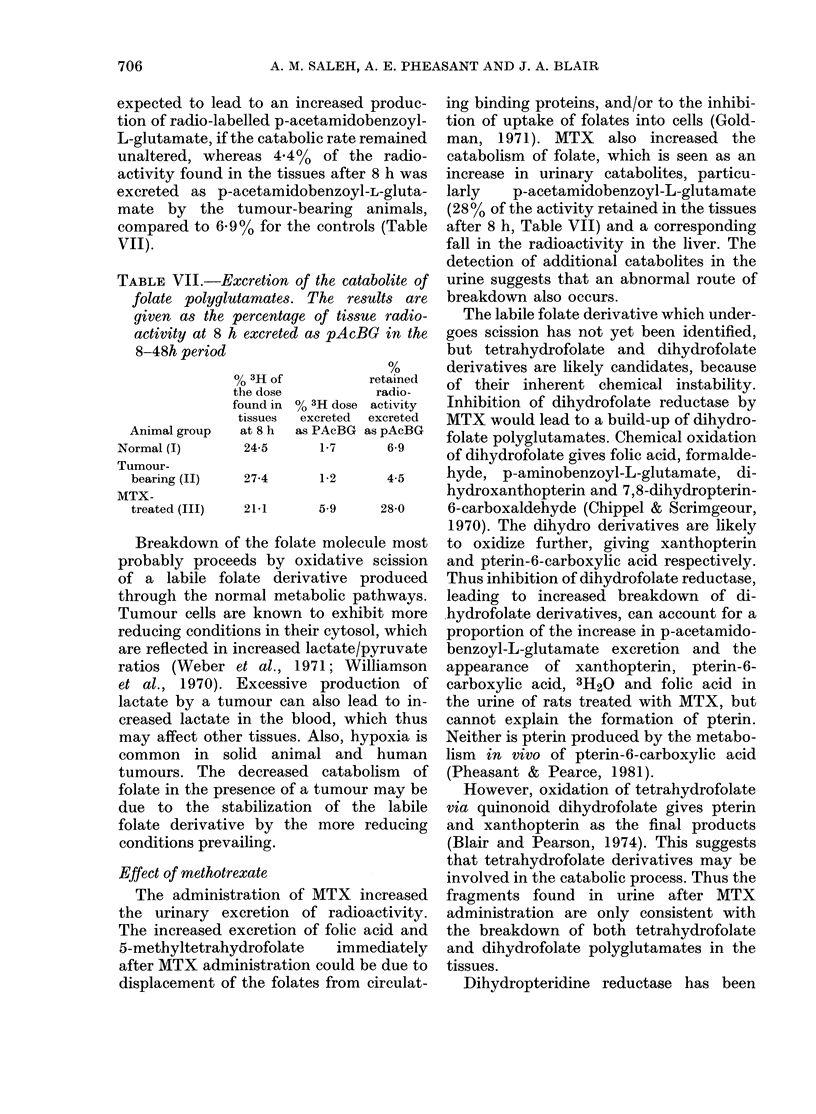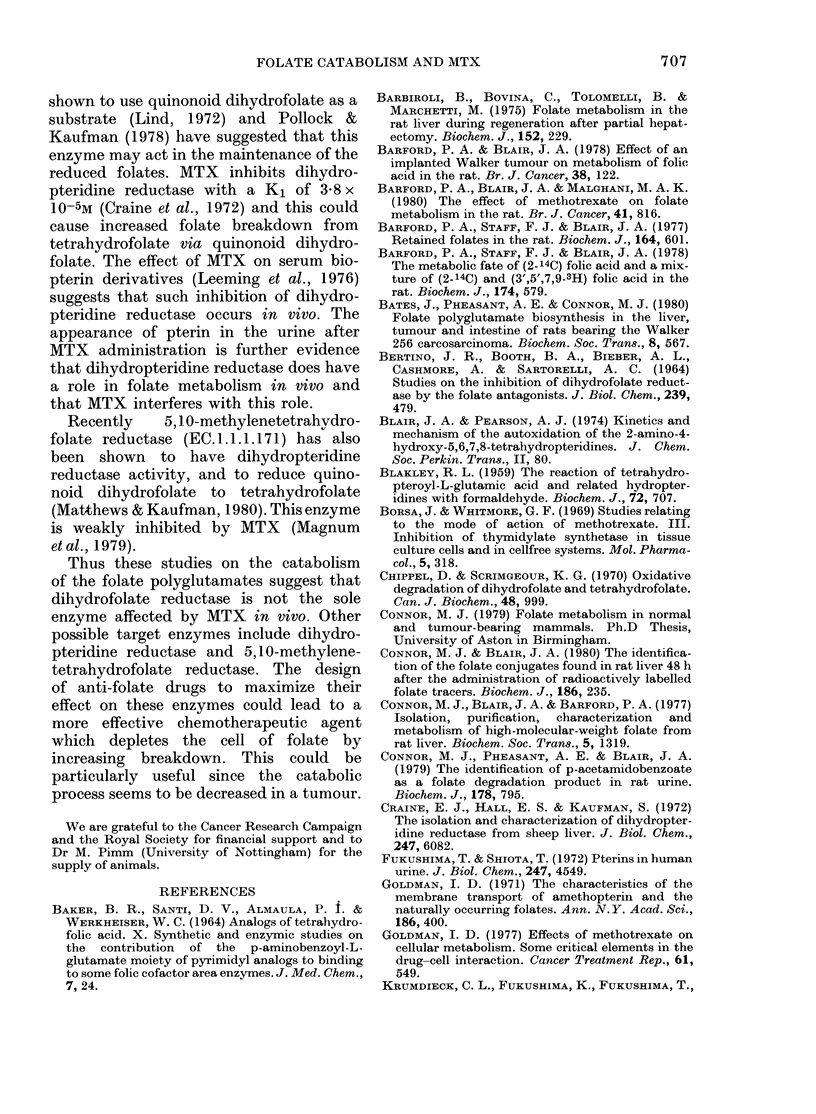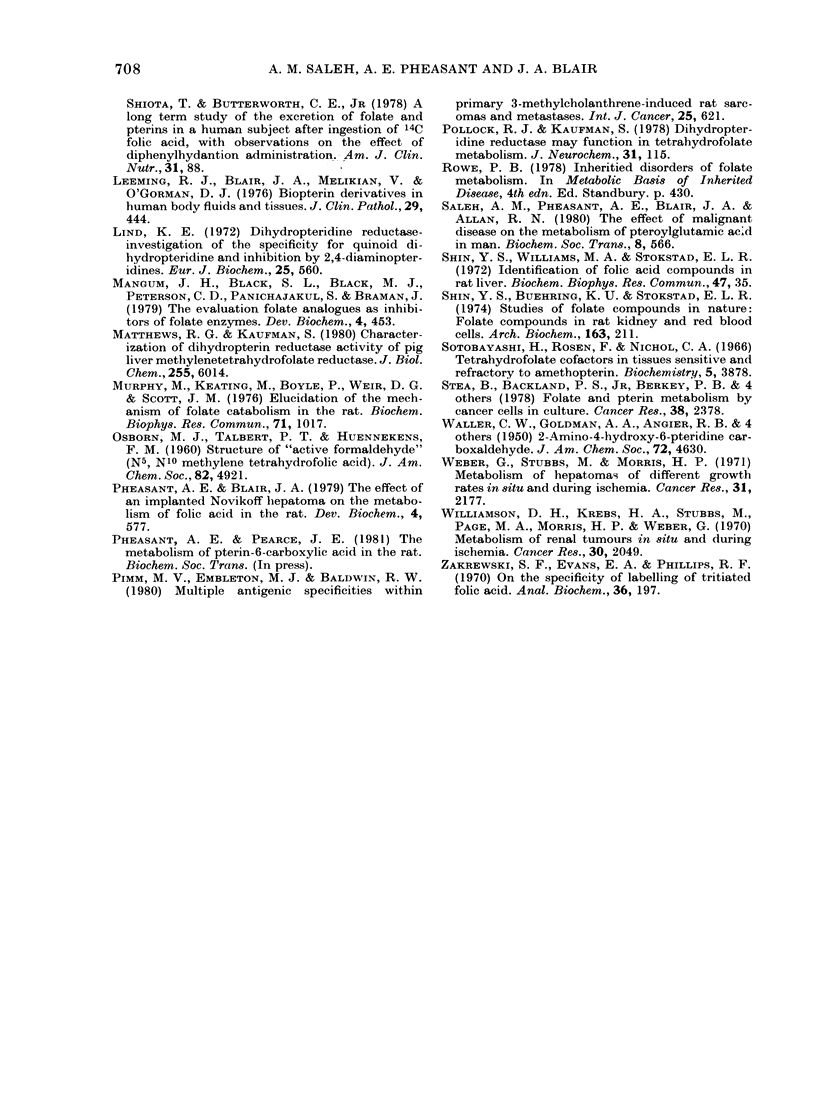# Folate catabolism in tumour-bearing rats and rats treated with methotrexate.

**DOI:** 10.1038/bjc.1981.256

**Published:** 1981-11

**Authors:** A. M. Saleh, A. E. Pheasant, J. A. Blair

## Abstract

The metabolism of (2-14C) + (3',5',7,9-3H) folic acid was studied in normal rats, tumour-bearing rats and rats treated with methotrexate (MTX). The experiments were designed to investigate changes in the catabolism and folate. The breakdown of folate to scission products was again demonstrated to be a normal phenomenon. Catabolites excreted included p-acetamidobenzoate, p-acetamidobenzoyl-L-glutamate, 3H2O, urea and a number of pterins. The catabolic process was decreased in the presence of a tumour and increased by the administration of MTX. MTX also led to the excretion of 4 additional radioactive pterins not found in normal urine. The possible mechanisms of folate breakdown are discussed with reference to the point of action of MTX.


*Br. J. Cancer* (1981) **44**, 700

### FOLATE CATABOLISM IN TUMOUR-BEARING RATS AND RATS TREATED WITH METHOTREXATE

A. M. SALEH, A. E. PHEASANT AND J. A. BLAIR

*From the Department of Chemistry, University of Aston in Birmingham, Birmingham B4 7ET*

Received 3 March 1981 Accepted 9 July 1981

**Summary.—The metabolism of (2-$^{14}$C) + (3′,5′,7,9-$^{3}$H) folic acid was studied in normal rats, tumour-bearing rats and rats treated with methotrexate (MTX). The experiments were designed to investigate changes in the catabolism of folate. The breakdown of folate to scission products was again demonstrated to be a normal phenomenon. Catabolites excreted included p-acetamidobenzoate, p-acetamidobenzoyl-L-glutamate, $^{3}H_2O$, urea and a number of pterins. The catabolic process was decreased in the presence of a tumour and increased by the administration of MTX. MTX also led to the excretion of 4 additional radioactive pterins not found in normal urine. The possible mechanisms of folate breakdown are discussed with reference to the point of action of MTX.**

IN RECENT YEARS there have been several reports of scission products appearing in urine as a result of folate degradation. p-Acetamidobenzoate (Connor *et al.*, 1979) and p-acetamidobenzoyl-L-glutamate (Murphy *et al.*, 1976) have been identified as catabolites of $^{3}$H folate tracers; pterin and isoxanthopterin have been detected after folic acid administration (Fukushima & Shiota, 1972; Krumdieck *et al.*, 1978) and Pheasant & Blair (1979) reported an unidentified pteridine as a metabolite of $^{3}$H- and $^{14}$C-folic acid.

A number of studies suggest that the pattern of folate coenzymes in malignant or normal rapidly dividing tissues is different from that of resting tissues (Sotobayashi *et al.*, 1966; Barbiroli *et al.*, 1975) and Stea *et al.* (1978) have claimed that tumour cells in culture produce 6-hydroxymethylpterin as a specific catabolite of folic acid. Folate metabolism *in vivo* is known to be affected by the presence of a tumour; preliminary studies suggest that the catabolism of folate may be depressed (Barford & Blair, 1978; Saleh *et al.*, 1980).

Methotrexate (MTX) is a folate antagonist which is used in cancer chemotherapy. It is a potent inhibitor of dihydrofolate reductase (EC.1.5.1.3, DHFR) (Bertino *et al.*, 1964) and is presumed to act by inhibiting this enzyme. However, the *in vivo* situation is complex, and a number of interacting mechanisms may be involved in MTX cytotoxicity (Goldman, 1977). The drug also inhibits other folate-dependent enzymes (*e.g.* thymidylate synthetase (EC.2.1.1.45) (Borsa & Whitmore, 1969) albeit at higher concentration) and acts on dihydropteridine reductase (EC.1.6.99.10) (Craine *et al.*, 1972) and Barford *et al.*, 1980) have presented evidence that DHFR is not the sole target of MTX methotrexate *in vivo*.

The major tissue folate derivatives are polyglutamyl conjugates (Shin *et al.*, 1972, 1974; Connor & Blair, 1980) and it appears likely that these derivatives function as coenzymes (Rowe, 1978). Previous studies have largely been concerned with the effect of MTX on the metabolism of radiolabelled folate monoglutamates. Administration of MTX, with or before folic acid, causes excretion of large amounts of unchanged folic acid, with little or no radio-label incorporated into the folate pool (Barford *et al.*, 1980). Effects on the normal tissue folates are therefore difficult to establish. The present study compares the metabolism of a mixture of 2-$^{14}$C-folic acid and 3′,5′,7,9-$^{3}$H-folic acid in normal rats, in rats treated with MTX and in tumour-bearing rats, the experiments involving MTX being designed to study its effect on folate polyglutamate metabolism, particularly their breakdown.

### MATERIALS AND METHODS

*Experimental design.*—Three groups of male syngeneic WAB/Not rats were used. Each group consisted of 15 rats.

Group I: Control rats (160–240 g body wt).

Group II: Rats bearing Mc103B sarcoma (260–320 g).

Group III: Normal rats (190–230 g) treated with MTX.

All rats received an oral dose of a mixture of 2-$^{14}$C- and 3′,5′,7,9-$^{3}$H-folic acid (100 $\mu$g/kg). Animals were then housed in metabolism cages (Jencons Metabowls, Jencons Scientific Ltd, Hemel Hempstead, Herts) designed for the separate collection of urine and faeces. Eight hours after the administration of labelled folic acid, 5 animals from each group were killed by cervical dislocation and the remaining animals of Group III were given an oral dose of MTX (100 mg/kg). Synthesis of folate polyglutamates in the tissues from labelled folic acid is complete by this time (Bates *et al.*, 1980). At 24 h a further 5 animals from each group were killed and the remainder of Group III received a second dose of MTX (100 mg/kg). All remaining animals were killed 24 h later. Liver, tumour, kidney and gut were removed for determination of radioactive content. Throughout all experiments animals were allowed food (Heygates Breeding Diet) and water *ad libitum.*

*Animals.*—Rats were supplied by Dr M. Pimm, University of Nottingham. The sarcoma Mc103B was induced in an adult male WAB/Not rat by s.c. injection of 1 mg 3-methylcholanthrene (Sigma, London) dissolved in 1 ml trioctanoin (Eastman Kodak, Rochester, N.Y., U.S.A.). A tumour transplant line was established and maintained in syngeneic male rats by s.c. trocar implantation of fragments of tumour tissue (Pimm *et al.*, 1980). For this experiment the sarcoma was implanted s.c. on the right flank and allowed to grow for 4 weeks.

*Collection of urine and faeces.*—Urine from 5 rats for each interval was collected into flasks containing 10 ml of 0·05M sodium phosphate buffer (pH 7·0) containing 2% (w/v) sodium ascorbate and 5 mg% (w/v) dithiothreitol. To prevent light degradation of folates the flasks were surrounded by aluminium foil. Collection flasks of urine and faeces were changed 8, 24 and 48 h after administration of labelled folic acid.

*Preparation of liver and tumour extracts.*—Hot extracts of livers and tumours were prepared as described by Barford *et al.* (1977).

*Determination of radioactivity.*—Urine samples and column effluents were counted as described in Connor *et al.* (1979). Faeces and tissue samples were freeze-dried and ground to give a homogeneous powder; 100mg samples were used to estimate total radioactivity, as described by Barford *et al.* (1978).

*Column chromatography.*—Sephadex G15 gel filtration and DEAE–cellulose chromatography (using linear gradients of 0–1·2M NaCl in 0·05M sodium phosphate buffer, pH 7·0) were performed as described by Barford *et al.* (1977).

Paper chromatography was performed as described by Connor *et al.* (1979).

*Chemicals.*—All chemicals used were of Analar grade or its equivalent. 2-$^{14}$C-folic acid (sp. radioact. 58 mCi/mmol) and 3′,5′,7,9-$^{3}$H-folic acid (sp. radioact. 500 mCi/mmol) were obtained from the Radiochemical Centre, Amersham, Bucks. p-Acetamidobenzoyl-L-glutamate was prepared as described in Baker *et al.* (1964), 10-formylfolate was synthesized from folic acid (Blakley, 1959), 5,10-methylene-tetrahydrofolate was prepared by Dr R. Nayyir Mazhir as described by Osborn *et al.* (1960), pterin-6-aldehyde-(Waller *et al.*, 1950) and pterin-6-carboxylic acid (Zakrewski *et al.*, 1970) were prepared by Dr M. Connor in this department.

### RESULTS

Table I summarizes the recovery of $^{3}$H and $^{14}$C radioactivity in the urine and faeces of the 3 groups of animals. More $^{3}$H than $^{14}$C was excreted in the urine of all animals, and an excess of $^{14}$C over $^{3}$H

TABLE I.—*Recovery of $^3H$ and $^{14}C$ in the urine and faeces of the 3 groups of animals given oral doses of a mixture of 2-$^{14}C$ and 3′,5′,7,9-$^3H$ folic acid (100 μg/kg). The results are expressed as the percentage of the dose recovered during the 3 collection periods (mean ± s.e., n = 5). Group I—Control rats; Group II—Tumour-bearing rats; Group III—Normal rats treated with methotrexate*

| Group No. | % of dose recovered 0–8 h $^3H$ | 0–8 h $^{14}C$ | 8–24 h $^3H$ | 8–24 h $^{14}C$ | 24–48 h $^3H$ | 24–48 h $^{14}C$ | Total $^3H$ | Total $^{14}C$ |
|---|---|---|---|---|---|---|---|---|
| Urine | | | | | | | | |
| Group I | 19·8 ± 2·6 | 16·7 ± 2·1 | 6·1 ± 0·9 | 3·8 ± 0·5 | 3·0 ± 0·3 | 2·1 ± 0·2 | 28·9 | 22·6 |
| Group II | 12·7 ± 2·4 | 9·9 ± 1·9 | 3·0 ± 0·5 | 2·1 ± 0·3 | 1·2 ± 0·1 | 1·0 ± 0·1 | 16·9 | 13·0 |
| Group III | 14·4 ± 1·6 | 11·7 ± 1·6 | 13·9 ± 1·3 | 11·4 ± 0·8 | 7·0 ± 0·4 | 5·6 ± 0·3 | 35·3 | 28·7 |
| Faeces | | | | | | | | |
| Group I | — | — | 11·0 ± 0·9 | 22·1 ± 1·9 | 4·8 ± 0·5 | 6·2 ± 0·9 | 15·8 | 28·3 |
| Group II | — | — | 3·4 ± 2·0 | 9·7 ± 5·2 | 0·9 ± 0·2 | 1·0 ± 0·2 | 4·3 | 10·7 |
| Group III | — | — | 5·3 ± 1·8 | 10·2 ± 3·9 | 0·7 ± 0·4 | 0·8 ± 0·5 | 6·0 | 11·0 |

was present in the faeces. No significant differences were observed in 0–8 h urinary radioactivity between control animals (taken as Group I + III) and tumour-bearing animals (Group II). In the later intervals the tumour-bearing rats excreted significantly less radioactivity in urine than control rats (Group I) ($P < 0{\cdot}02$ for 8–24 h; $0{\cdot}001 < P < 0{\cdot}01$ for 24–48 h) whereas MTX increased the radioactivity appearing in the 8–24 h and 24–48 h urine samples of the Group III animals ($P < 0{\cdot}001$ for both intervals). These results reflect differences in the metabolism of folate polyglutamate derivatives.

Faecal recovery of radioactivity was also reduced in Groups II and III but this was due to non-production of faeces by a number of rats.

TABLE II.—*The chromatographic properties of the various metabolites appearing in the urine of the 3 groups of rats*

| Compound* | Elution position: Sephadex G15 gel filtration (2 × 60 cm) fraction No. (5ml fractions) | DEAE–cellulose chromatography (2 × 40 cm) [NaCl] (M) |
|---|---|---|
| Folic acid | 37 | 0·96 |
| 5Me THF | 37 | 0·67 |
| 10CHOFA | 21 | 0·53 |
| 5,10CH₂THF | 25 | 0·64 |
| Folate (X) | 36 | 0·70 |
| p-AcBA | 36 | 0·43 |
| p-AcBG | 19 | 0·43 |
| Metabolite A | 41 | 0·40 |
| Metabolite B | 54 | 0·32 |
| Pterin | 35 | 0·30 |
| pterin-6-COOH | 30 | 0·60 |
| Xanthopterin | 57 | 0·57 |
| Metabolite C | 42 | 0·60 |
| $^3H_2O$ | 21 | 0·0 |
| Urea | 21 | 0·0 |

* 5Me THF = 5-methyltetrahydrofolate;
10CHOFA = 10-formylfolate;
5,10$CH_2$THF = 5,10-methylenetetrahydrofolate;
pAcBA = p-acetamidobenzoate;
p-AcBG = p-acetamidobenzoyl-L-glutamate;
pterin-6-COOH = pterin-6-carboxylic acid.

*Urinary metabolites*

Urine samples were subjected to sequential chromatography on DEAE–cellulose and Sephadex G15. This revealed a complex mixture of 11 metabolites appearing in the urine of control and tumour-bearing rats, and at least 14 metabolites were detected in the urine of the Group III animals after MTX administration. The chromatographic behaviour of the various metabolites is given in Table II, and Tables III–V show the relative distribution of each metabolite appearing in the various urine samples.

The same metabolites were seen in urine from rats in Groups I and II, though in differing amounts. Five intact folates were excreted; folic acid, 5-methyltetrahydrofolate, 10-formylfolate and 5,10-methylenetetrahydrofolate were identified by co-chromatography with authentic standards in both column sys-

TABLE III.—*Metabolites present in the urine of the control rats after the administration of 2-$^{14}C$ and 3′,5′,7,9-$^{3}H$-folic acid* (100 $\mu g/kg$)

| | % dose recovered as each metabolite | | | | | | | |
|---|---|---|---|---|---|---|---|---|
| | 0–8 h | | 8–24 h | | 24–48 h | | Total | |
| Metabolite* | $^{3}H$ | $^{14}C$ | $^{3}H$ | $^{14}C$ | $^{3}H$ | $^{14}C$ | $^{3}H$ | $^{14}C$ |
| Folic acid | 1·4 | 1·3 | 0·1 | 0·1 | trace | trace | 1·5 | 1·4 |
| 5MeTHF | 6·9 | 6·5 | 0·9 | 0·6 | 0·4 | 0·4 | 8·2 | 7·5 |
| 10CHOFA | 5·8 | 5·1 | 1·4 | 0·9 | 0·6 | 0·4 | 7·8 | 6·4 |
| 5,10$CH_2$THF | 0·9 | 0·8 | 0·3 | 0·2 | — | — | 1·2 | 1·0 |
| Folate (X) | — | — | 0·5 | 0·4 | — | — | 0·5 | 0·4 |
| p-AcBA | 1·7 | — | 1·5 | — | 0·6 | — | 3·8 | — |
| p-AcBG | 2·0 | — | 0·7 | — | 1·0 | — | 3·7 | — |
| Metabolite A† | + | 1·4 | + | 0·7 | + | 0·7 | + | 2·8 |
| Metabolite B | 0·2 | 1·0 | 0·1 | 0·5 | 0·1 | 0·3 | 0·4 | 1·8 |
| $^{3}H_2O$ | 0·4 | — | 0·2 | — | 0·3 | — | 0·9 | — |
| Urea | — | 0·6 | — | 0·2 | — | 0·2 | — | 1·0 |

* See footnote to Table II.
† The positive $^{3}H$ label associated with Metabolite A could not be estimated.

TABLE IV.—*Metabolites present in the urine of tumour-bearing rats following the administration of 2-$^{14}C$ and 3′,5′,7,9-$^{3}H$-folic acid* (100 $\mu g/kg$)

| | % dose recovered as each metabolite | | | | | | | |
|---|---|---|---|---|---|---|---|---|
| | 0–8 h | | 8–24 h | | 24–48 h | | Total | |
| Metabolite | $^{3}H$ | $^{14}C$ | $^{3}H$ | $^{14}C$ | $^{3}H$ | $^{14}C$ | $^{3}H$ | $^{14}C$ |
| Folic acid | 4·2 | 3·5 | 0·4 | 0·4 | trace | 0·1 | 4·6 | 4·0 |
| 5MeTHF | 3·2 | 2·7 | 0·3 | 0·3 | 0·1 | 0·1 | 3·6 | 3·1 |
| 10CHOFA | 1·8 | 1·5 | 0·4 | 0·3 | — | — | 2·2 | 1·8 |
| 5,10$CH_2$THF | 0·6 | 0·5 | — | — | — | — | 0·6 | 0·5 |
| Folate (X) | 0·7 | 0·7 | 0·1 | 0·1 | — | — | 0·8 | 0·8 |
| p-AcBA | 0·7 | — | 0·7 | — | 0·3 | — | 1·7 | — |
| p-AcBG | 1·3 | — | 0·7 | — | 0·5 | — | 2·5 | — |
| Metabolite A† | + | 0·6 | + | 0·6 | + | 0·5 | + | 1·7 |
| Metabolite B | 0·1 | 0·4 | 0·1 | 0·2 | trace | 0·1 | 0·2 | 0·7 |
| $^{3}H_2O$ | 0·1 | — | 0·2 | — | 0·2 | — | 0·5 | — |
| Urea | — | 0·1 | — | 0·1 | — | 0·1 | — | 0·3 |

† The positive $^{3}H$ label associated with Metabolite A could not be estimated.

TABLE V.—*Metabolites present in the urine of normal rats after the administration of 2-$^{14}C$ and 3′,5′,7,9-$^{3}H$-folic acid* (100 $\mu g/kg$) *and MTX* (100 *mg/kg body wt*) *after* 8 *h and* 24 *h*

| | % dose recovered as each metabolite | | | | | | | |
|---|---|---|---|---|---|---|---|---|
| | 0–8 h | | 8–24 h | | 24–48 h | | Total | |
| Metabolite | $^{3}H$ | $^{14}C$ | $^{3}H$ | $^{14}C$ | $^{3}H$ | $^{14}C$ | $^{3}H$ | $^{14}C$ |
| Folic acid | 0·7 | 0·7 | 2·0 | 1·8 | 0·7 | 0·9 | 3·4 | 3·4 |
| 5MeTHF | 6·0 | 5·1 | 2·6 | 2·3 | 0·4 | 0·5 | 9·0 | 7·9 |
| 10CHOFA | 5·3 | 4·2 | 1·7 | 1·4 | 0·6 | 0·4 | 7·6 | 6·0 |
| 5,10$CH_2$THF | 0·7 | 0·6 | 0·7 | 0·7 | — | — | 1·4 | 1·3 |
| p-AcBA | 0·5 | — | 2·2 | — | 0·7 | — | 3·4 | — |
| p-AcBG | 0·5 | — | 2·9 | — | 3·0 | — | 6·4 | — |
| Metabolite A† | + | 0·9 | + | 1·0 | + | 0·5 | + | 2·4 |
| Metabolite B | 0·1 | 0·4 | 0·1 | 0·7 | 0·1 | 0·5 | 0·3 | 1·6 |
| Metabolite C | — | — | 0·1 | 0·2 | 0·2 | 0·5 | 0·3 | 0·7 |
| Pterin | — | — | 0·1 | 0·7 | trace | 0·3 | 0·1 | 1·0 |
| Pterin-6-COOH† | — | — | + | 1·4 | + | 0·7 | + | 2·1 |
| Xanthopterin | — | — | 0·2 | 0·7 | 0·1 | 0·6 | 0·3 | 1·3 |
| $^{3}H_2O$ | 0·2 | — | 0·4 | — | 0·7 | — | 1·3 | — |
| Urea | — | 0·2 | — | 0·2 | — | 0·3 | — | 0·7 |

† The positive $^{3}H$ label associated with Metabolite A and Pterin-6-COOH could not be estimated.

TABLE VI.—*Recovery of radioactivity in the tissues of 3 groups of rats receiving oral doses of a mixture of* 2-$^{14}$*C and* 3′,5′,7,9-$^{3}$*H-folic acid. The results are expressed as percentage of the dose* (*mean* ± *s.e.*, n = 5)

| | % dose radioactivity recovered | | | | | | | | | | | | | | | | | | | | |
|---|---|---|---|---|---|---|---|---|---|---|---|---|---|---|---|---|---|---|---|---|---|
| | I. Control | | | | | | | II. Tumour-bearer | | | | | | | III. Normal rats + MTX | | | | | | |
| | Tissue wt | 8 h | | 24 h | | 48 h | | Tissue wt | 8 h | | 24 h | | 48 h | | Tissue wt | 8 h | | 24 h | | 48 h | |
| Tissue | (g) | $^{3}$H | $^{14}$C | $^{3}$H | $^{14}$C | $^{3}$H | $^{14}$C | (g) | $^{3}$H | $^{14}$C | $^{3}$H | $^{14}$C | $^{3}$H | $^{14}$C | (g) | $^{3}$H | $^{14}$C | $^{3}$H | $^{14}$C | $^{3}$H | $^{14}$C |
| Liver | 7·8 ±0·4 | 16·4 ±0·7 | 17·5 ±0·8 | 18·1 ±0·7 | 19·1 ±0·7 | 19·1 ±0·6 | 20·7 ±0·5 | 9·2 ±0·3 | 13·4 ±1·1 | 12·6 ±1·0 | 16·8 ±0·9 | 15·7 ±0·9 | 16·5 ±1·3 | 16·8 ±1·3 | 8·0 ±0·4 | 14·9 ±1·2 | 13·9 ±1·3 | 13·6 ±9·9 | 13·9 ±0·4 | 12·0 ±0·7 | 12·4 ±0·9 |
| Tumour | — | — | — | — | — | — | — | 18·9 ±1·4 | 7·9 ±0·9 | 8·6 ±1·2 | 9·3 ±1·1 | 9·3 ±1·0 | 7·0 ±1·0 | 7·6 ±1·2 | — | — | — | — | — | — | — |
| Gut | 5·5 ±0·3 | 4·8 ±0·4 | 4·5 ±0·5 | 3·0 ±0·2 | 2·7 ±0·2 | 2·3 ±0·06 | 2·0 ±0·07 | 5·7 ±0·2 | 2·8 ±0·3 | 2·7 ±0·3 | 2·7 ±0·3 | 2·5 ±0·2 | 2·0 ±0·2 | 1·7 ±0·2 | 5·2 ±0·2 | 3·7 ±0·3 | 3·5 ±0·3 | 2·4 ±0·1 | 1·9 ±0·1 | 1·0 ±0·1 | 0·8 ±0·1 |
| Kidney | 1·9 ±0·1 | 3·3 ±0·1 | 3·3 ±0·2 | 1·7 ±0·1 | 1·8 ±0·1 | 1·6 ±0·06 | 1·6 ±0·05 | 2·3 ±0·1 | 3·3 ±0·4 | 3·0 ±0·4 | 1·6 ±0·1 | 1·6 ±0·1 | 1·6 ±0·1 | 1·6 ±0·1 | | 2·5 ±0·2 | 2·1 ±0·2 | 1·4 ±0·1 | 1·2 ±0·1 | 1·1 ±0·1 | 1·0 ±0·1 |
| Total | | 24·5 | 25·3 | 22·8 | 23·6 | 23·0 | 24·3 | | 27·4 | 26·9 | 30·4 | 29·1 | 27·1 | 27·7 | | 21·1 | 19·5 | 17·4 | 17·0 | 14·1 | 14·2 |

tems (the fifth intact folate (folate X) has not yet been identified). Tumour-bearing animals excreted less of the dose as folate in the urine. Rats in Groups I and II excreted 6 catabolites: 3 of these were labelled solely with $^3H$ and were identified as p-acetamidobenzoate, p-acetamidobenzoyl-L-glutamate and $^3H_2O$; one catabolite was labelled solely with $^{14}C$, and may be urea (Connor *et al.*, 1977) and 2 metabolites (A and B) had an increased $^{14}C$: $^3H$ ratio and were possibly pteridines. Excretion of all scission products was decreased in the tumour-bearing rats.

MTX increased considerably the excretion of folic acid, 5-methyltetrahydrofolate and a number of catabolites, in particular p-acetamidobenzoyl-L-glutamate. In addition to increasing excretion of normal catabolites, MTX also led to the production of 4 additional radioactive scission products. None of these was detected in the urine of rats not receiving MTX and all had $^{14}C$:$^3H$ ratios suggesting pteridines. Three of these additional metabolites were identified as pterin, pterin-6-carboxylic acid and xanthopterin by co-chromatography with authentic standards in both column systems. We were unable to identify the fourth metabolite (C).

*Liver and tumour extracts*

Chromatography on Sephadex G15 of supernatants of liver extracts prepared from the 3 groups of animals and of tumour extracts prepared from Group II gave similar results. In all cases the major radioactive peak eluted in the position of the folate polyglutamates (*i.e.* close to the void volume). Small amounts of single-labelled compounds (including pterin) were present, but no folate monoglutamates could be detected at any time.

*Recovery of radioactivity in tissues*

Table VI shows the radioactivity retained in the tissues of all groups of animals at various intervals. In control and tumour-bearing rats the radioactivity in the liver increased gradually with time, whereas MTX appeared to cause a fall in hepatic radioactivity. Considerable amounts of radioactivity were present in the tumour tissue, thus increasing the proportion of the dose retained in the tumour-bearing rats.

### DISCUSSION

These studies again demonstrate that considerable catabolism of the folate molecule into non-folate scission products is a normal phenomenon. The rate at which this breakdown occurs could have a significant effect on the folate status of the animal.

*Effect of a tumour*

Tumour-bearing rats excreted less radioactivity in both the urine and faeces. Qualitatively this is due to a lowered excretion of most metabolites in the urine. Although excretion of unchanged folic acid was higher than normal, overall excretion of folates was reduced. This is probably due to uptake of folate and formation of polyglutamates by a large tumour mass. Similar observations have been reported for human subjects suffering from malignancies (Saleh *et al.*, 1980). Catabolism of folate was also decreased in the tumour-bearing rats. The catabolites appearing after the initial 8 h period arise principally from the breakdown of folate polyglutamates, since there was no evidence of folate monoglutamates in the tissue extracts. p-Acetamidobenzoyl-L-glutamate is derived by breakdown of the tissue polyglutamates, whereas p-acetamidobenzoate is the catabolite of the monoglutamate pool (Connor, 1979). The levels of p-acetamidobenzoyl-L-glutamate can therefore be used as a measure of the breakdown of tissue polyglutamate. Excretion of this compound in the urine is decreased in the tumour-bearing animals. This observation is particularly striking if the increased radioactivity in the tissues of the tumour-bearing animals is taken into account. The larger amounts of radiolabelled polyglutamates would be expected to lead to an increased production of radio-labelled p-acetamidobenzoyl-L-glutamate, if the catabolic rate remained unaltered, whereas 4·4% of the radioactivity found in the tissues after 8 h was excreted as p-acetamidobenzoyl-L-glutamate by the tumour-bearing animals, compared to 6·9% for the controls (Table VII).

TABLE VII.—*Excretion of the catabolite of folate polyglutamates. The results are given as the percentage of tissue radioactivity at 8 h excreted as pAcBG in the 8–48h period*

| Animal group | % $^3$H of the dose found in tissues at 8 h | % $^3$H dose excreted as PAcBG | % retained radioactivity excreted as pAcBG |
|---|---|---|---|
| Normal (I) | 24·5 | 1·7 | 6·9 |
| Tumour-bearing (II) | 27·4 | 1·2 | 4·5 |
| MTX-treated (III) | 21·1 | 5·9 | 28·0 |

Breakdown of the folate molecule most probably proceeds by oxidative scission of a labile folate derivative produced through the normal metabolic pathways. Tumour cells are known to exhibit more reducing conditions in their cytosol, which are reflected in increased lactate/pyruvate ratios (Weber *et al.*, 1971; Williamson *et al.*, 1970). Excessive production of lactate by a tumour can also lead to increased lactate in the blood, which thus may affect other tissues. Also, hypoxia is common in solid animal and human tumours. The decreased catabolism of folate in the presence of a tumour may be due to the stabilization of the labile folate derivative by the more reducing conditions prevailing.

*Effect of methotrexate*

The administration of MTX increased the urinary excretion of radioactivity. The increased excretion of folic acid and 5-methyltetrahydrofolate immediately after MTX administration could be due to displacement of the folates from circulating binding proteins, and/or to the inhibition of uptake of folates into cells (Goldman, 1971). MTX also increased the catabolism of folate, which is seen as an increase in urinary catabolites, particularly p-acetamidobenzoyl-L-glutamate (28% of the activity retained in the tissues after 8 h, Table VII) and a corresponding fall in the radioactivity in the liver. The detection of additional catabolites in the urine suggests that an abnormal route of breakdown also occurs.

The labile folate derivative which undergoes scission has not yet been identified, but tetrahydrofolate and dihydrofolate derivatives are likely candidates, because of their inherent chemical instability. Inhibition of dihydrofolate reductase by MTX would lead to a build-up of dihydrofolate polyglutamates. Chemical oxidation of dihydrofolate gives folic acid, formaldehyde, p-aminobenzoyl-L-glutamate, dihydroxanthopterin and 7,8-dihydropterin-6-carboxaldehyde (Chippel & Scrimgeour, 1970). The dihydro derivatives are likely to oxidize further, giving xanthopterin and pterin-6-carboxylic acid respectively. Thus inhibition of dihydrofolate reductase, leading to increased breakdown of dihydrofolate derivatives, can account for a proportion of the increase in p-acetamidobenzoyl-L-glutamate excretion and the appearance of xanthopterin, pterin-6-carboxylic acid, $^3H_2O$ and folic acid in the urine of rats treated with MTX, but cannot explain the formation of pterin. Neither is pterin produced by the metabolism *in vivo* of pterin-6-carboxylic acid (Pheasant & Pearce, 1981).

However, oxidation of tetrahydrofolate *via* quinonoid dihydrofolate gives pterin and xanthopterin as the final products (Blair and Pearson, 1974). This suggests that tetrahydrofolate derivatives may be involved in the catabolic process. Thus the fragments found in urine after MTX administration are only consistent with the breakdown of both tetrahydrofolate and dihydrofolate polyglutamates in the tissues.

Dihydropteridine reductase has been shown to use quinonoid dihydrofolate as a substrate (Lind, 1972) and Pollock & Kaufman (1978) have suggested that this enzyme may act in the maintenance of the reduced folates. MTX inhibits dihydropteridine reductase with a $K_I$ of $3{\cdot}8 \times 10^{-5}$M (Craine *et al.*, 1972) and this could cause increased folate breakdown from tetrahydrofolate *via* quinonoid dihydrofolate. The effect of MTX on serum biopterin derivatives (Leeming *et al.*, 1976) suggests that such inhibition of dihydropteridine reductase occurs *in vivo*. The appearance of pterin in the urine after MTX administration is further evidence that dihydropteridine reductase does have a role in folate metabolism *in vivo* and that MTX interferes with this role.

Recently 5,10-methylenetetrahydrofolate reductase (EC.1.1.1.171) has also been shown to have dihydropteridine reductase activity, and to reduce quinonoid dihydrofolate to tetrahydrofolate (Matthews & Kaufman, 1980). This enzyme is weakly inhibited by MTX (Magnum *et al.*, 1979).

Thus these studies on the catabolism of the folate polyglutamates suggest that dihydrofolate reductase is not the sole enzyme affected by MTX *in vivo*. Other possible target enzymes include dihydropteridine reductase and 5,10-methylenetetrahydrofolate reductase. The design of anti-folate drugs to maximize their effect on these enzymes could lead to a more effective chemotherapeutic agent which depletes the cell of folate by increasing breakdown. This could be particularly useful since the catabolic process seems to be decreased in a tumour.

We are grateful to the Cancer Research Campaign and the Royal Society for financial support and to Dr M. Pimm (University of Nottingham) for the supply of animals.

REFERENCES

Baker, B. R., Santi, D. V., Almaula, P. I. & Werkheiser, W. C. (1964) Analogs of tetrahydrofolic acid. X. Synthetic and enzymic studies on the contribution of the p-aminobenzoyl-L-glutamate moiety of pyrimidyl analogs to binding to some folic cofactor area enzymes. *J. Med. Chem.*, **7**, 24.

Barbiroli, B., Bovina, C., Tolomelli, B. & Marchetti, M. (1975) Folate metabolism in the rat liver during regeneration after partial hepatectomy. *Biochem. J.*, **152**, 229.

Barford, P. A. & Blair, J. A. (1978) Effect of an implanted Walker tumour on metabolism of folic acid in the rat. *Br. J. Cancer*, **38**, 122.

Barford, P. A., Blair, J. A. & Malghani, M. A. K. (1980) The effect of methotrexate on folate metabolism in the rat. *Br. J. Cancer*, **41**, 816.

Barford, P. A., Staff, F. J. & Blair, J. A. (1977) Retained folates in the rat. *Biochem. J.*, **164**, 601.

Barford, P. A., Staff, F. J. & Blair, J. A. (1978) The metabolic fate of (2-$^{14}$C) folic acid and a mixture of (2-$^{14}$C) and (3′,5′,7,9-$^{3}$H) folic acid in the rat. *Biochem. J.*, **174**, 579.

Bates, J., Pheasant, A. E. & Connor, M. J. (1980) Folate polyglutamate biosynthesis in the liver, tumour and intestine of rats bearing the Walker 256 carcosarcinoma. *Biochem. Soc. Trans.*, **8**, 567.

Bertino, J. R., Booth, B. A., Bieber, A. L., Cashmore, A. & Sartorelli, A. C. (1964) Studies on the inhibition of dihydrofolate reductase by the folate antagonists. *J. Biol. Chem.*, **239**, 479.

Blair, J. A. & Pearson, A. J. (1974) Kinetics and mechanism of the autoxidation of the 2-amino-4-hydroxy-5,6,7,8-tetrahydropteridines. *J. Chem. Soc. Perkin. Trans.*, II, 80.

Blakley, R. L. (1959) The reaction of tetrahydropteroyl-L-glutamic acid and related hydropteridines with formaldehyde. *Biochem. J.*, **72**, 707.

Borsa, J. & Whitmore, G. F. (1969) Studies relating to the mode of action of methotrexate. III. Inhibition of thymidylate synthetase in tissue culture cells and in cellfree systems. *Mol. Pharmacol.*, **5**, 318.

Chippel, D. & Scrimgeour, K. G. (1970) Oxidative degradation of dihydrofolate and tetrahydrofolate. *Can. J. Biochem.*, **48**, 999.

Connor, M. J. (1979) Folate metabolism in normal and tumour-bearing mammals. Ph.D Thesis, University of Aston in Birmingham.

Connor, M. J. & Blair, J. A. (1980) The identification of the folate conjugates found in rat liver 48 h after the administration of radioactively labelled folate tracers. *Biochem. J.*, **186**, 235.

Connor, M. J., Blair, J. A. & Barford, P. A. (1977) Isolation, purification, characterization and metabolism of high-molecular-weight folate from rat liver. *Biochem. Soc. Trans.*, **5**, 1319.

Connor, M. J., Pheasant, A. E. & Blair, J. A. (1979) The identification of p-acetamidobenzoate as a folate degradation product in rat urine. *Biochem. J.*, **178**, 795.

Craine, E. J., Hall, E. S. & Kaufman, S. (1972) The isolation and characterization of dihydropteridine reductase from sheep liver. *J. Biol. Chem.*, **247**, 6082.

Fukushima, T. & Shiota, T. (1972) Pterins in human urine. *J. Biol. Chem.*, **247**, 4549.

Goldman, I. D. (1971) The characteristics of the membrane transport of amethopterin and the naturally occurring folates. *Ann. N.Y. Acad. Sci.*, **186**, 400.

Goldman, I. D. (1977) Effects of methotrexate on cellular metabolism. Some critical elements in the drug–cell interaction. *Cancer Treatment Rep.*, **61**, 549.

Krumdieck, C. L., Fukushima, K., Fukushima, T.,

SHIOTA, T. & BUTTERWORTH, C. E., JR (1978) A long term study of the excretion of folate and pterins in a human subject after ingestion of $^{14}C$ folic acid, with observations on the effect of diphenylhydantion administration. *Am. J. Clin. Nutr.*, **31**, 88.

LEEMING, R. J., BLAIR, J. A., MELIKIAN, V. & O'GORMAN, D. J. (1976) Biopterin derivatives in human body fluids and tissues. *J. Clin. Pathol.*, **29**, 444.

LIND, K. E. (1972) Dihydropteridine reductase-investigation of the specificity for quinoid dihydropteridine and inhibition by 2,4-diaminopteridines. *Eur. J. Biochem.*, **25**, 560.

MANGUM, J. H., BLACK, S. L., BLACK, M. J., PETERSON, C. D., PANICHAJAKUL, S. & BRAMAN, J. (1979) The evaluation folate analogues as inhibitors of folate enzymes. *Dev. Biochem.*, **4**, 453.

MATTHEWS, R. G. & KAUFMAN, S. (1980) Characterization of dihydropterin reductase activity of pig liver methylenetetrahydrofolate reductase. *J. Biol. Chem.*, **255**, 6014.

MURPHY, M., KEATING, M., BOYLE, P., WEIR, D. G. & SCOTT, J. M. (1976) Elucidation of the mechanism of folate catabolism in the rat. *Biochem. Biophys. Res. Commun.*, **71**, 1017.

OSBORN, M. J., TALBERT, P. T. & HUENNEKENS, F. M. (1960) Structure of "active formaldehyde" ($N^5$, $N^{10}$ methylene tetrahydrofolic acid). *J. Am. Chem. Soc.*, **82**, 4921.

PHEASANT, A. E. & BLAIR, J. A. (1979) The effect of an implanted Novikoff hepatoma on the metabolism of folic acid in the rat. *Dev. Biochem.*, **4**, 577.

PHEASANT, A. E. & PEARCE, J. E. (1981) The metabolism of pterin-6-carboxylic acid in the rat. *Biochem. Soc. Trans.* (In press).

PIMM, M. V., EMBLETON, M. J. & BALDWIN, R. W. (1980) Multiple antigenic specificities within primary 3-methylcholanthrene-induced rat sarcomas and metastases. *Int. J. Cancer*, **25**, 621.

POLLOCK, R. J. & KAUFMAN, S. (1978) Dihydropteridine reductase may function in tetrahydrofolate metabolism. *J. Neurochem.*, **31**, 115.

ROWE, P. B. (1978) Inheritied disorders of folate metabolism. In *Metabolic Basis of Inherited Disease*, *4th edn*. Ed. Standbury. p. 430.

SALEH, A. M., PHEASANT, A. E., BLAIR, J. A. & ALLAN, R. N. (1980) The effect of malignant disease on the metabolism of pteroylglutamic acid in man. *Biochem. Soc. Trans.*, **8**, 566.

SHIN, Y. S., WILLIAMS, M. A. & STOKSTAD, E. L. R. (1972) Identification of folic acid compounds in rat liver. *Biochem. Biophys. Res. Commun.*, **47**, 35.

SHIN, Y. S., BUEHRING, K. U. & STOKSTAD, E. L. R. (1974) Studies of folate compounds in nature: Folate compounds in rat kidney and red blood cells. *Arch. Biochem.*, **163**, 211.

SOTOBAYASHI, H., ROSEN, F. & NICHOL, C. A. (1966) Tetrahydrofolate cofactors in tissues sensitive and refractory to amethopterin. *Biochemistry*, **5**, 3878.

STEA, B., BACKLAND, P. S., JR, BERKEY, P. B. & 4 others (1978) Folate and pterin metabolism by cancer cells in culture. *Cancer Res.*, **38**, 2378.

WALLER, C. W., GOLDMAN, A. A., ANGIER, R. B. & 4 others (1950) 2-Amino-4-hydroxy-6-pteridine carboxaldehyde. *J. Am. Chem. Soc.*, **72**, 4630.

WEBER, G., STUBBS, M. & MORRIS, H. P. (1971) Metabolism of hepatomas of different growth rates *in situ* and during ischemia. *Cancer Res.*, **31**, 2177.

WILLIAMSON, D. H., KREBS, H. A., STUBBS, M., PAGE, M. A., MORRIS, H. P. & WEBER, G. (1970) Metabolism of renal tumours *in situ* and during ischemia. *Cancer Res.*, **30**, 2049.

ZAKREWSKI, S. F., EVANS, E. A. & PHILLIPS, R. F. (1970) On the specificity of labelling of tritiated folic acid. *Anal. Biochem.*, **36**, 197.